# Forms and Amounts of Vitamin B12 in Infant Formula: A Pilot Study

**DOI:** 10.1371/journal.pone.0165458

**Published:** 2016-11-16

**Authors:** Eva Greibe, Ebba Nexo

**Affiliations:** Department of Clinical Biochemistry, Aarhus University Hospital, Aarhus, Denmark; Bauer Research foundation, UNITED STATES

## Abstract

**Purpose:**

Infant formula is based on cow’s milk and designed to mimic breast milk for substitution. Vitamin B12 (B12) is bound to proteins in both breast milk and cow’s milk, and in milk from both species the vitamin occurs mainly in its natural form such as hydroxo-B12 with little or no synthetic B12 (cyano-B12). Here we test commercially available infant formulas.

**Methods:**

Eleven commercially available infant formulas were measured for content of B12 and analyzed for the presence of B12-binding proteins and forms of B12 using size exclusion chromatography and HPLC.

**Results:**

All infant formulas contained B12 by and large in accord with the informations given on the package inserts. None of the formulas contained protein-bound B12, and cyano-B12 accounted for 19–78% of the total amount of B12 present, while hydroxo-B12 constituted more or less the rest.

**Conclusions:**

This pilot study shows that infant formula differs from breast milk in providing the infant with free B12, rather than protein-bound B12, and by a relative high content of cyano-B12. The consequence of supplying the infant with synthetic cyano-B12 remains to be elucidated.

## Introduction

Vitamin B12 (B12, cobalamin) is important during early development, and the new-born child depends on breast milk or infant formula for supply of the vitamin. In breast milk, virtually all B12 is bound to the B12-binding protein haptocorrin (previously named transcobalamin I) [1, 2]. The function of haptocorrin in breast milk is unknown, but a role in early infant B12 absorption has been proposed [3–5]. In adults, B12 absorption depends on the presence of gastric acid, digestive enzymes, and the gastrointestinal transport protein, intrinsic factor (reviewed by [6]). Intrinsic factor binds B12 and facilitates the transport over the intestinal wall by binding to its receptor, Cubam, in the terminal ileum [6]. In neonates, the concentrations of pepsin, gastric acid, and intrinsic factor in the gastrointestinal tract is low [5, 7, 8], possibly due to a yet immature absorption system. This has lead scientists to speculate if early B12-absorption occurs—at least in part—through an intrinsic factor-independent mechanism, and if the unexplained high concentrations of haptocorrin in breast milk mediate B12 absorption in the first weeks of life by acting as stand-in for an immature absorption system (outlined by [5]).

Infant formula is based on cow’s milk and designed to mimic breast milk for substitution. However, in contrast to breast milk, cow’s milk does not contain haptocorrin, and B12 is bound to another transport protein, transcobalamin (previously named transcobalamin II) [9].

Different forms of B12 exist. Vitamin pills usually contain the stable and synthetic form, cyano-B12; whereas food items including breast milk and cow’s milk mostly contain hydroxo-B12, methyl-B12 and 5’-deoxyadenosyl-B12 [10, 11]. Methyl-B12 and 5´-deoxyadenosyl-B12 are the active forms of B12 acting as coenzymes for the methionine synthase in the conversion of homocysteine to methionine in the cytoplasm and for the methylmalonyl-CoA mutase in the conversion of methylmalonyl-CoA to succinyl-CoA in the mitochondrion, respectively. All forms of B12 are converted into the coenzymes in the cell before metabolized [12]. Inborn errors affecting the intracellular processing of B12 into the coenzyme forms can be devastating in early life causing severe B12 deficiency. Interestingly, children who fail to produce methyl-B12 and 5’-deoxyadenosyl-B12 respond better to treatment with hydroxo-B12 compared with cyano-B12 (summarized by [12]). There is no knowledge on the content of the various B12 forms in infant formulas.

In this study, we test 11 commercially available infant formulas for content of B12 and its binding proteins and determine the content of natural and synthetic B12.

## Materials and Methods

### Infant formula

Eleven commercially available infant formulas from four different manufacturers were chosen for the study and purchased from three Danish supermarkets during fall 2015. The supermarkets were Foetex in Storcenter Nord, Aarhus (location: 56.170463,10.188628), Loevbjerg at Troejborg, Aarhus (location: 56.172469,10.211981), and Kvickly in Aabyhoej, Aarhus (location: 56.155764,10.164378). An overview of the products is presented in Table 1 together with the main results. Six of the products were categorized as breast milk substitutes by the manufacturers and thereby designed to provide a 0–6 month infant with a complete diet. The other five products were follow-on formulas designed to supplement a wholesome diet (Table 1). The products from Nestlé and HIPP are available in many parts of the world, including United States and countries in Latin America, Asia, and Europe. The products from Sember and Arla are mainly sold in Scandinavia.

**Table 1:** Results on 11 infant formulas.

		Indicated /100 ml		Observed /100 ml				
No	Product name and (status)	Protein g	B12 μg	Total B12 μg	CN-B12 μg (%)	HO-B12 μg (%)	Ado-B12 μg (%)	CH_3_-B12 μg (%)
**Semper:**								
**1**	Allomin 1 (BMS)	1.40	0.16	0.22	0.06 (25)	0.11 (50)	0.03 (15)	0.02 (10)
**2**	Allomin 2 (FOF)	1.60	0.22	0.24	0.07 (29)	0.17 (71)	0.00 (0)	0.00 (0)
**Nestlé:**								
**3**	NAN1 (BMS)	1.24	0.15	0.17	0.05 (32)	0.11 (64)	0.009 (5)	0.00 (0)
**4**	NAN H.A.1 (BMS)	1.27	0.14	0.25	0.20 (78)	0.04 (17)	0.01 (4)	0.003 (1)
**5**	NAN2 (FOF)	1.34	0.19	0.21	0.13 (63)	0.08 (37)	0.00 (0)	0.00 (0)
**6**	NAN3 (FOF)	1.50	0.22	0.20	0.13 (63)	0.04 (21)	0.03 (13)	0.006 (3)
**Arla:**								
**7**	Baby & Me 1 (BMS)	1.40	0.25	0.16	0.05 (31)	0.08 (52)	0.00 (0)	0.03 (17)
**8**	Babymaelk (BMS)	1.55	0.19	0.18	0.10 (57)	0.08 (43)	0.00 (0)	0.00 (0)
**9**	Baby & Me 2 (FOF)	2.00	0.23	0.23	0.09 (40)	0.09 (40)	0.02 (8)	0.01 (4)
**HIPP:**								
**10**	Baby Combiotic 1 (BMS)	1.40	0.50	0.34	0.23 (69)	0.11 (31)	0.00 (0)	0.00 (0)
**11**	Baby Combiotic 2 (FOF)	1.50	0.15	0.21	0.04 (19)	0.09 (42)	0.008 (4)	0.07 (35)
**Breast milk and cow’s milk:**								
**-**	Breast milk at 4 month postpartum [1, 11]	-	-	0.04	(< 5)	(~40)^1^	(~40)^1^	(~60)
-	Cow’s milk, (skimmed)[9, 11]	-	-	0.45	(< 5)	(~100)^1^	(~100)^1^	(< 5)

Status as breast milk substitute (BMS) (from birth to 6 months of age) or follow-on formula (FOF) (from 6 months of age and onwards) is provided, and so is the B12 and protein content as indicated on the package inserts by the manufacturers (indicated/100 ml of dissolved/liquid product). Measured values for total B12 are presented (observed/100 ml of dissolved/liquid product). Calculated contents and (percentages) of the synthetic cyano-B12 (CN-B12) and natural hydroxo-B12 (HO-B12), 5´-deoxyadenosyl-B12 (Ado-B12) and methyl-B12 (CH_3_-B12) are indicated. Data for breast milk [1, 11] and cow’s milk [9, 11] is shown for comparison. ^1^HO-B12 and Ado-B12 was measured together and are given as joint percentages.

All infant formulas but one (no. 4) were liquid ready-to-use products. The containers were shaken, opened in red dim light, and the milky content was transferred to light-protective vials. All products appeared normal and uncontaminated upon opening and looked and smelled as would be expected for infant formula. Infant formula no. 4 was purchased as a milk powder and dissolved as indicated by the manufacturer (one measuring spoon (approx. 6 g) powder in 30 ml of 40˚C tap water). Also this procedure was carried out in red dim light. All formulas were analyzed for content of B12 and for the presence of B12-binding proteins and forms of B12 (see below).

### Determination of B12

Total B12 was measured using the Cobas 6000 E immunoassay system (Roche Diagnostics, Hvidovre, Denmark) with a measurement range of 55–1476 pM. The infant formula was diluted 1:10 in 0.9% NaCl solution prior to analysis.

### Determination of B12-binding proteins

**Fig 1:**
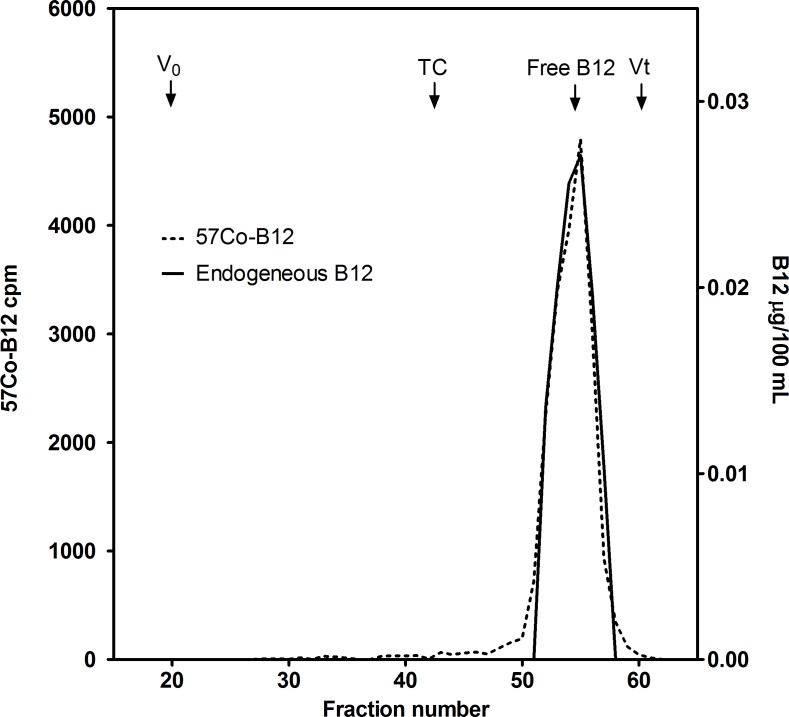
Size exclusion chromatography of B12 in infant formula no. 3 (NAN1). The x-axis indicates fraction number. Elution volume for void volume (V_0_), transcobalamin (TC), free B12, and total volume (V_t_) are indicated. B12 was on a free form, and no B12 eluted together with the elution profile of transcobalamin or other B12-binding proteins. The graphics were created in Graph Pad Prism version 5.

The presence of B12-binding proteins was explored by size exclusion chromatography. Two ml infant formula was centrifuged at 16000 g for 10 minutes at room temperature, and 1 ml of the supernatant was incubated with 100 μl 5 nM radiolabelled B12 (^57^[Co]-cobalamin) (Kem-En-Tec, Taastrup, Denmark) for 30 minutes at room temperature. Then, 500 μl of the mixture was applied to a Superdex 200 column (GE Healthcare, Broendby, Denmark) attached to a Dionex ICS-3000 HPLC system and was eluted with a flow rate of 400 μL/minute for approximately 70 minutes employing a Tris buffer (0.1 mol/L) (Sigma Aldrich, Broendby, Denmark), 1 mol/L NaCl (Merck A/S Denmark, Hellerup, Denmark), 0.5 g/L bovine albumin (Sigma Aldrich), 0.2 g/L sodium-azide, pH 8. Fractions of 400 μl were collected and were measured for radioactivity in the Wizard Automatic Gamma Counter (Perkin Elmer) and for B12 content in the Cobas 6000 E immunoassay system. Blue Dextran (Sigma-Aldrich, Broendby, Denmark) and Na^22^ (GE Healthcare, Broendby, Denmark) was used for determination of void volume (V_0_) and total volume (V_t_), respectively. Results are presented in Fig 1 and S1 Fig.

### Determination of B12 forms

**Fig 2:**
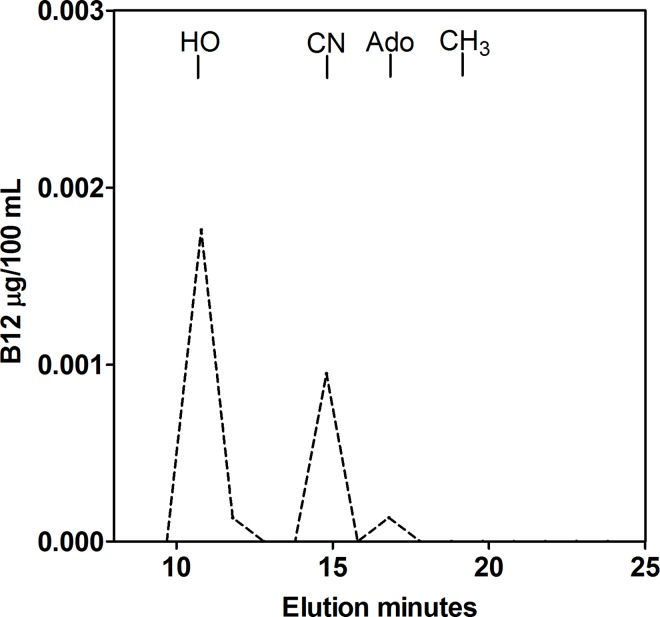
The forms of B12 in infant formula no. 3 (NAN1). B12 was extracted in the dark and subjected to HPLC. B12 in each post-column fraction was measured by an in-house ELISA. The positions of elution for hydroxo-B12 (HO), cyano-B12 (CN), 5’-deoxyadenosyl-B12 (Ado), and methyl-B12 (CH_3_) are indicated. B12 eluted predominantly as hydroxo-B12 and cyano-B12 in all infant formulas tested. The graphics were created in Graph Pad Prism version 5.

B12 forms were measured essentially as described by Hardlei et al [13]. In brief, in dim red light 500 μl infant formula was incubated with 500 μl 0.4M acetic acid and 1000 μl 50% methanol for 15 minutes at 65°C before centrifugation for 3 minutes at 11000 g through a 0.22 μm Durepor PVDF filter (Merck Millipore Ltd., Ireland). Ninety μl of the filtered sample was injected into an Agilent 1260 Infinity HPLC (Agilent Technologies, Germany) attached to a reverse-phase column (Luna 3u reverse-phase C18(2) 150 mm x 4.6 mm, Phenomenex) and run with a flow rate of 1 ml/minute. A gradient of acetonitrile (HPLC Sgrade, Rathburn Chemicals, United Kingdom) increasing from 5% to 30% over 20 minutes in 0.010 mol/L phosphoric acid (H3PO4, pH 3) was applied 4 minutes after injection. Standards with pure hydroxo-B12 (GEA, Copenhagen, Denmark), cyano-B12 (Sigma Aldrich, Germany), methyl-B12 (Sigma Aldrich, Germany) and 5’deoxyadenosyl-B12 (Sigma Aldrich, Germany) were used to identify the elution times of hydroxo-B12 (10.8 minutes), cyano-B12 (14.8 minutes), 5’-deoxyadenosyl-B12 (16.8 minutes), and methyl-B12 (19.8 minutes). Due to the low amounts of B12 present in the samples, we could not measure the B12 elution profile by recording OD. Instead we collected 15 one-minute’s post-column fractions (1000 μL/fraction) between 9 and 23 minutes after injection. The samples were lyophilized and dissolved in 240 μL PBA-NaOH buffer (120 μl 2M NaOH in 4000 μl 0.1% phosphate buffered albumin) prior to estimating the content of B12. In order to achieve a precise estimate for B12, we used a previously described in-house haptocorrin ELISA [13, 14] with a detection limit of 8 pmol/L B12 rather than the Cobas assay that has a detection limit of 55 pmol/L B12. In brief, 100 μl calibrators or samples were incubated with apo-haptocorrin. After removal of excess apo-haptocorrin with B12-coated magnetic beads, the amount of B12 saturated haptocorrin present in the supernatant was measured by ELISA. A calibration curve was drawn from the included calibrators (0–218 pmol/L), and the results for the samples were red from the curve. The assay signal was linear for B12 concentrations between 0 and 218 pmol/L and the total imprecision (CV) was ≤ 10% (measured for B12 concentrations between 45 and 200 pmol/L). The results for the B12 measures were used to construct the elution profiles presented in Fig 2 and S2 Fig and for calculating the fractional content of each form present, as indicated in Table 1. The absolute amount of each form of the vitamin was calculated by multiplying the fractional content with the total B12 concentration in the infant formula, as measured by the Cobas assay. A HPLC chromatogram is exemplified in S3 Fig.

## Results

We examined 11 commercially available infant formulas for their content and forms of B12. The results are presented in Table 1.

We performed size exclusion chromatography and showed all B12 present in the breast milk substitute formulas to elute as free B12. In addition, the results showed absence of B12-binding proteins, since all added labeled B12 also eluted in its free form. The results are exemplified in Fig 1. The same elution pattern was observed for all infant formulas tested (S1 Fig).

We also investigated the forms of B12 using HPLC. These studies were carried out in red dim light to avoid the conversion of methyl-B12 and 5’-deoxyadenosyl-B12 to hydroxo-B12 upon light exposure. For 10 out of 11 products, virtually all B12 was present either as hydroxo-B12 or cyano-B12, and only trace amounts of methyl-B12 and 5’-deoxyadenosyl-B12 were detected. One product (No. 11) did contain a substantial amount of methyl-B12 (35%). The percentage of cyano-B12 out of total B12 was between 19% and 78% (see Table 1). The results are exemplified in Fig 2. The HPLC profiles of the other ten infant formulas can be seen in S2 Fig.

## Discussion

We explored the B12 content in 11 infant formulas; six breast milk substitutes and five follow-on formulas, and determined the content of B12-binding proteins and forms of B12.

The most surprising result was that five infant formulas (No. 4, 5, 6, 8 and 10) contained substantial amounts of cyano-B12, ranging from 57% to 78%, while both breast milk and cow’s milk contains mainly hydroxo-B12. Currently the clinical outcome of using one or the other form of B12 is unknown. However recent rat studies question whether the two forms of B12 is of equal value. After an acute dose of hydroxo-B12 more than twice as much B12 accumulates in the liver than after administration of an acute dose of cyano-B12 [15]. In contrast, cyano-B12 induces the most pronounced increase in plasma B12 [15]. If this result mirrors the situation in infants it suggests that administration of cyano-B12 may improve the circulating level of B12 but only to a limited degree ensure tissue uptake of the vitamin.

Our measurements of B12 contents in the infant formulas did not perfectly agree with the information given on the packaging (Table 1, Indicated versus Observed). However, when taking uncertainty of measurement and differences in methods into account, the variation is to be expected. The concentration of B12 in infant formula ((median [range]) 0.21 [0.16–0.34] μg/100 mL) is about twice as high as in breast milk collected at two weeks postpartum (0.10 [0.03–0.26] μg/100 mL) (n = 25, Danish women) and five times higher than in breast milk collected at four month postpartum (0.04 [0.02–0.09] μg/100 mL) (n = 25, Danish women) [1]. In contrast, cow’s milk (mean ± SD) (0.45 ± 0.04 μg/100 mL) contain about twice as much B12 as infant formula [9]. According to the information given on the package inserts, children aged 0–6 month should consume 700–1000 mL infant formula per day. Using the median B12 concentration (0.21 μg/100 mL), we estimate the daily amount of B12 ingested from infant formula to be 1.5–2.1 μg. In comparison, The Recommended Dietary Allowance for children aged 0–6 month is 0.4 μg B12 [16].

The finding of free B12 in breast milk substitute infant formula strongly suggests that transcobalamin and other possible B12-binding compounds in cow’s milk have been destroyed during preparation of the infant formula. The importance of transcobalamin binding of B12 in milk is yet to be determined, but transcobalamin is believed to ensure the high bioavailability of B12 from milk [17–19]. This potential positive effect of transcobalamin on B12 absorption from cow’s milk raises the question if infant formula would be a better source of B12 if processing is performed in a manner that do not destroy transcobalamin.

Clinical studies comparing infant formula and breast milk with relation to infant B12 status, as judged from Specker et al, 1990 [20], suggest that infant formula is at least as efficient as breast milk in ensuring a healthy infant B12 status. Therefore one can argue that the relatively high amounts of B12 present in formula is as efficient as supplying the child with the smaller amount of protein-bound B12 present in breast milk. However, this conclusion may have to be modified. We do not know to which extent the tested formula contained cyano-B12, and because of that presently we do not know whether it is high amounts of free hydroxo-B12 or cyano-B12, or either that will ensure a sufficient B12 status in the infant.

## Conclusion

This pilot study shows that infant formula differs from breast milk and cow’s milk in providing the infant with free B12, rather than B12 bound to haptocorrin (breast milk) or transcobalamin (cow’s milk). In some infant formulas, the major part of B12 is present as cyano-B12 rather than as the naturally occurring forms of the vitamin. The consequence of supplying the infant with free cyano-B12 as compared to protein-bound natural B12 remains to be elucidated, and so does the composition of B12 forms in various commercially available infant formulas from around the world.

## Supporting Information

S1 Fig Size exclusion chromatography of B12 in infant formula The horizontal axes indicate fraction number. Elution volume for void volume (V_0_), transcobalamin (TC), free B12, and total volume (V_t_) are indicated. B12 was on a free form, and no B12 eluted together with the elution profile of transcobalamin or other B12-binding proteins. The same elution pattern was observed for all infant formulas tested. The graphics were created in Graph Pad Prism version 5
(TIF)

S2 Fig Analysis of the forms of B12 in infant formula. B12 was extracted in the dark and subjected to HPLC. B12 in each post-column fraction was measured by an in-house ELISA as described in materials and methods. The positions of elution for hydroxo-B12 (HO), cyano-B12 (CN), 5’-deoxyadenosyl-B12 (Ado), and methyl-B12 (CH_3_) are indicated. B12 was found to be predominantly hydroxo-B12 and cyano-B12 in all infant formulas tested. The graphics were created in Graph Pad Prism version 5
(TIF)

S3 Fig HPLC chromatogram. The 11 infant formulas were subjected to HPLC in order to separate the different forms of B12 present in the products. The column used was a Luna 3u reverse-phase C18(2) 150 mm x 4.6 mm (Phenomenex) attached to a Agilent 1260 Infinity HPLC (Agilent Technologies, Germany). Ninety μl standards (pure hydroxo-B12, cyano-B12, methyl-B12, and 5’-deoxyadenosyl-B12) or samples were run with a gradient of acetonitrile (Solvent C) increasing from 5% to 30% over 20 minutes in 0.010 M phosphoric acid (Solvent A). Paracetamol was used as an internal standard. Fifteen one-minute’s post-column fractions were collected from each run (starting at 9 minutes) and analysed for its content of B12 by an in-house ELISA. For details on the ELISA, see the method section in the main paper. Elusion times for hydroxo-B12, cyano-B12, methyl-B12, and 5’-deoxyadenosyl-B12 was 10.8 minutes, 14.8 minutes, 16.8 minutes, and 19.8 minutes, respectively. The peaks are not visible at the chromatogram since the amount of B12 present in the samples and standards were far below the detection limit of OD measurements. OD visible amounts of B12 standards were avoided in order not to contaminate the column. Four graphs are shown, all with the X-axis in minutes. Upper graph shows the elution profile (OD 254 nm) for the internal standard (peak at 10.097 minutes) and signals indicating change of fraction. The two middle graphs indicate the solvent concentrations (%) of phosphoric acid (Solvent A) and acetonitrile (Solvent C). The lower graph indicates column pressure (bar).
(PDF)
