# Supplementary figures and images for: Forms and Amounts of Vitamin B12 in Infant Formula: A Pilot Study
Source: PLoS One. 2016 Nov 16;11(11):e0165458. doi: 10.1371/journal.pone.0165458 (PMC5112923; doi:10.1371/journal.pone.0165458)

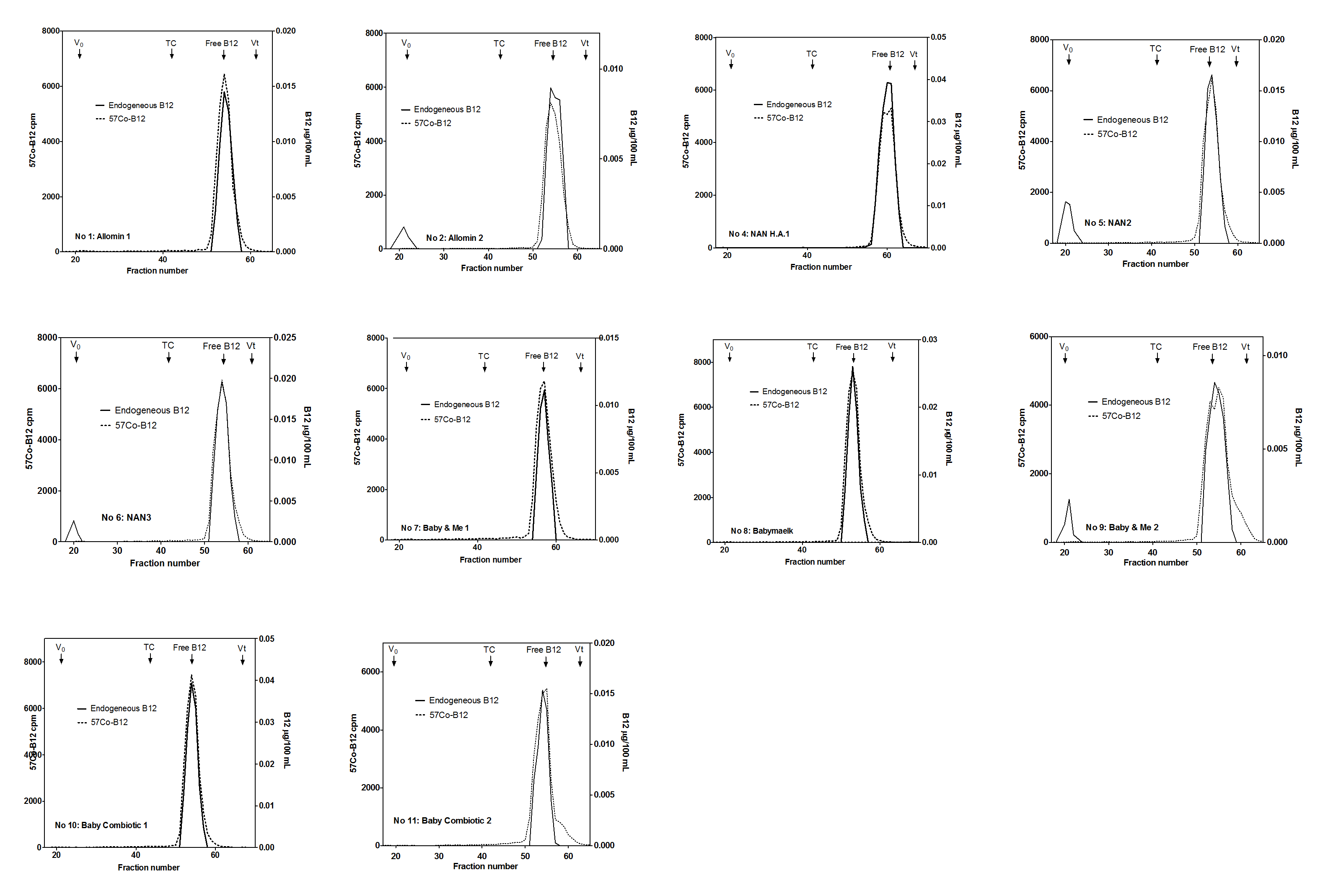

Supplement: S1 Fig — Elution volume for void volume (V0), transcobalamin (TC), free B12, and total volume (Vt) are indicated. B12 was on a free form, and no B12 eluted together with the elution profile of transcobalamin or other B12-binding proteins. The same elution pattern was observed for all infant formulas tested. The graphics were created in Graph Pad Prism version 5 (TIF) [file pone.0165458.s001.tif]

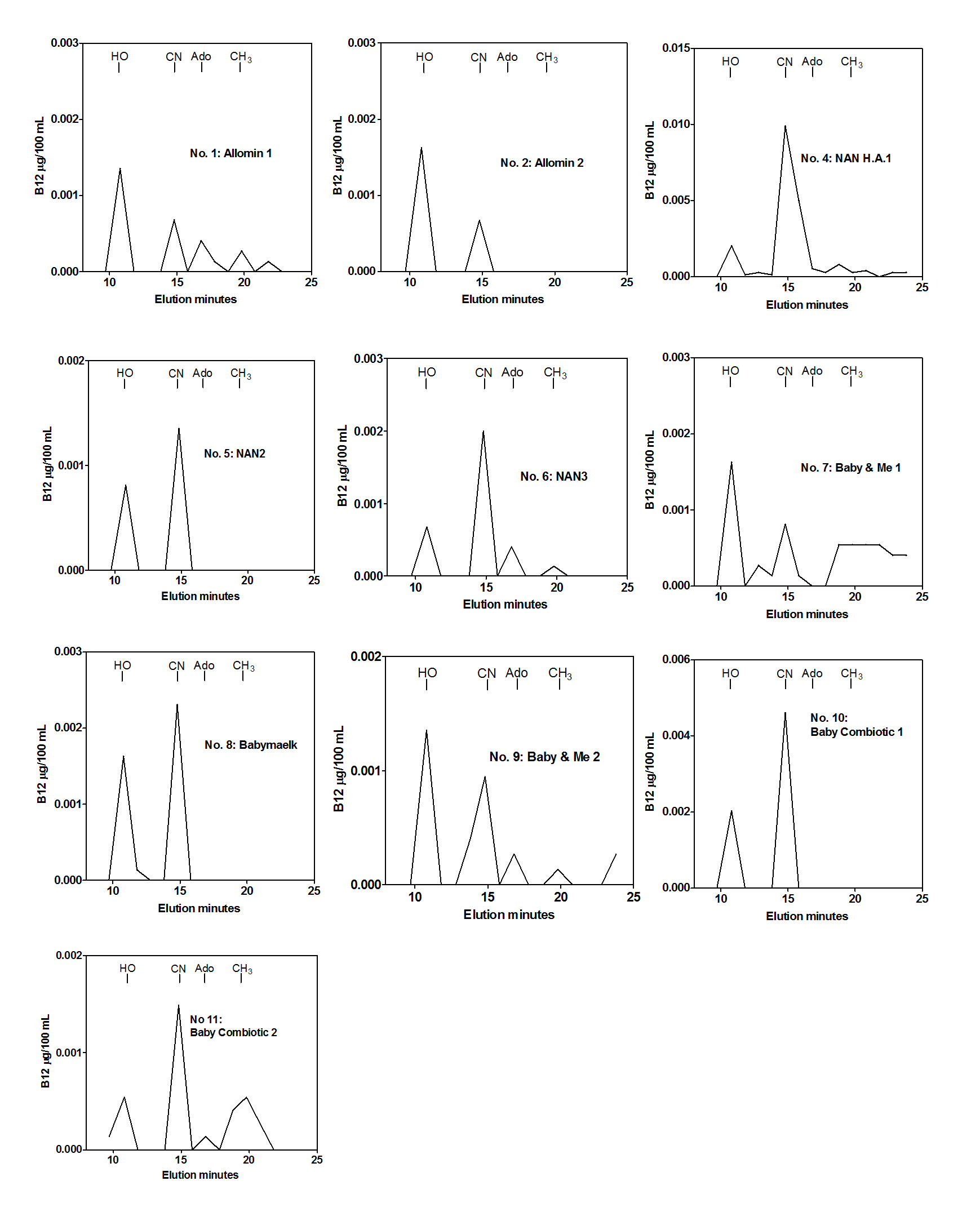

Supplement: S2 Fig — B12 was extracted in the dark and subjected to HPLC. B12 in each post-column fraction was measured by an in-house ELISA as described in materials and methods. The positions of elution for hydroxo-B12 (HO), cyano-B12 (CN), 5’-deoxyadenosyl-B12 (Ado), and methyl-B12 (CH3) are indicated. B12 was found to be predominantly hydroxo-B12 and cyano-B12 in all infant formulas tested. The graphics were created in Graph Pad Prism version 5 (TIF) [file pone.0165458.s002.tif]
