# Supplementary material for: Forms and Amounts of Vitamin B12 in Infant Formula: A Pilot Study
Source: PLoS One. 2016 Nov 16;11(11):e0165458. doi: 10.1371/journal.pone.0165458 (PMC5112923; doi:10.1371/journal.pone.0165458)
Supplement: S3 Fig — The 11 infant formulas were subjected to HPLC in order to separate the different forms of B12 present in the products. The column used was a Luna 3u reverse-phase C18(2) 150 mm x 4.6 mm (Phenomenex) attached to a Agilent 1260 Infinity HPLC (Agilent Technologies, Germany). Ninety μl standards (pure hydroxo-B12, cyano-B12, methyl-B12, and 5’-deoxyadenosyl-B12) or samples were run with a gradient of acetonitrile (Solvent C) increasing from 5% to 30% over 20 minutes in 0.010 M phosphoric acid (Solvent A). Paracetamol was used as an internal standard. Fifteen one-minute’s post-column fractions were collected from each run (starting at 9 minutes) and analysed for its content of B12 by an in-house ELISA. For details on the ELISA, see the method section in the main paper. Elusion times for hydroxo-B12, cyano-B12, methyl-B12, and 5’-deoxyadenosyl-B12 was 10.8 minutes, 14.8 minutes, 16.8 minutes, and 19.8 minutes, respectively. The peaks are not visible at the chromatogram since the amount of B12 present in the samples and standards were far below the detection limit of OD measurements. OD visible amounts of B12 standards were avoided in order not to contaminate the column. Four graphs are shown, all with the X-axis in minutes. Upper graph shows the elution profile (OD 254 nm) for the internal standard (peak at 10.097 minutes) and signals indicating change of fraction. The two middle graphs indicate the solvent concentrations (%) of phosphoric acid (Solvent A) and acetonitrile (Solvent C). The lower graph indicates column pressure (bar). (PDF) [file pone.0165458.s003.pdf]

Acq. Operator : jfi
Acq. Instrument : Instrument 1 Location : Vial 1
Injection Date : 7/11/2016 1:11:15 PM
Inj Volume : 25 µl
Acq. Method : C:\CHEM32\1\METHODS\AC-SEPARATION AF 4 B12 VARIANTER 9 - 24.M
Last changed : 7/11/2016 1:09:31 PM by jfi
(modified after loading)
Analysis Method : C:\CHEM32\1\METHODS\AC-SEPARATION AF 4 B12 VARIANTER 9 - 24.M
Last changed : 7/11/2016 1:47:22 PM by jfi
(modified after loading)

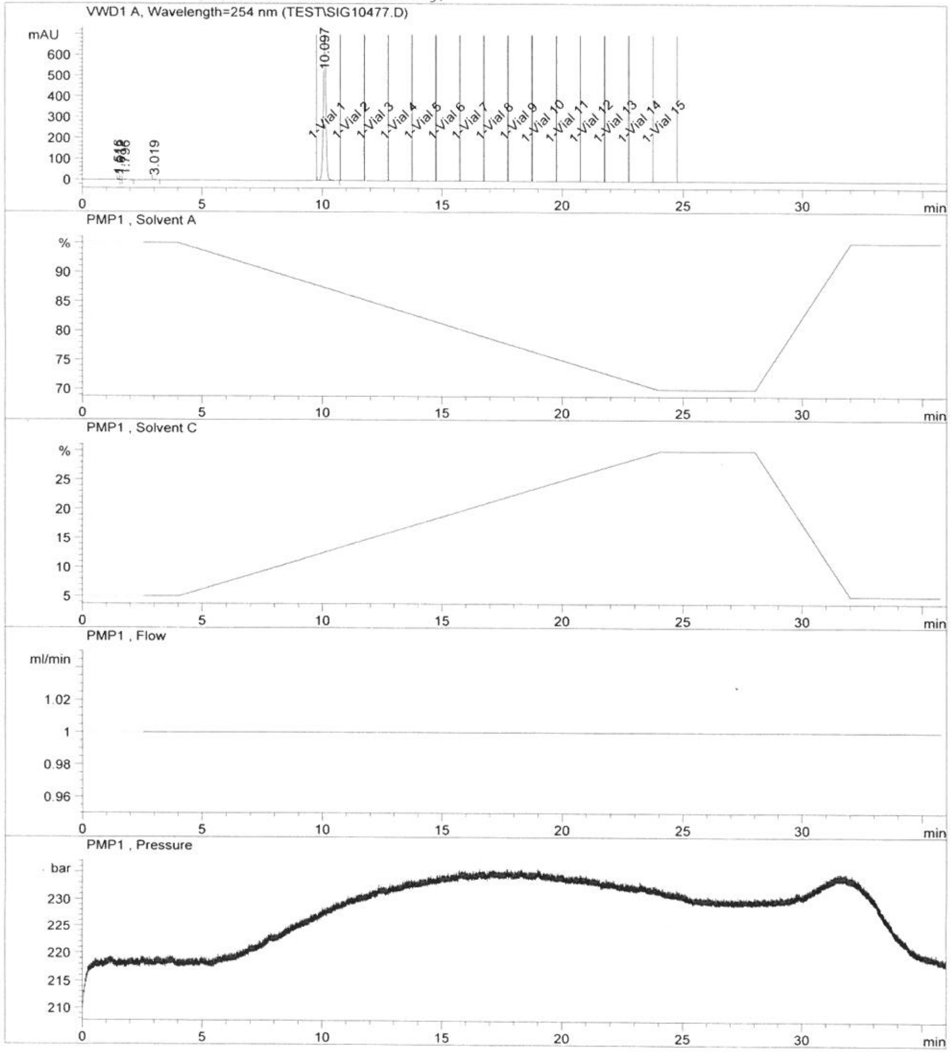

Fraction Information

Data File C:\CHEM32\1\DATA\TEST\SIG10477.D
Sample Name: 110716 kontrol CN

Fraction collection using a timetable

Frac #	Well #	Location	Volume [µl]	BeginTime [min]	EndTime [min]	Reason	Mass
1	1	1-Vial 1	1000.21	9.7498	10.7500	Time	
2	1	1-Vial 2	988.75	10.7613	11.7500	Time	
3	1	1-Vial 3	988.75	11.7613	12.7500	Time	
4	1	1-Vial 4	991.46	12.7586	13.7500	Time	
5	1	1-Vial 5	992.71	13.7573	14.7500	Time	
6	1	1-Vial 6	992.08	14.7580	15.7500	Time	
7	1	1-Vial 7	992.71	15.7573	16.7500	Time	
8	1	1-Vial 8	988.75	16.7613	17.7500	Time	
9	1	1-Vial 9	989.38	17.7607	18.7500	Time	
10	1	1-Vial 10	988.12	18.7621	19.7502	Time	
11	1	1-Vial 11	966.04	19.7840	20.7500	Time	
12	1	1-Vial 12	991.46	20.7586	21.7500	Time	
13	1	1-Vial 13	992.08	21.7579	22.7500	Time	
14	1	1-Vial 14	992.50	22.7575	23.7500	Time	
15	1	1-Vial 15	992.71	23.7573	24.7500	Time	

Area Percent Report

Sorted By : Signal
Multiplier : 1.0000
Dilution : 1.0000
Use Multiplier & Dilution Factor with ISTDs

Signal 1: VWD1 A, Wavelength=254 nm

Peak #	RetTime [min]	Type	Width [min]	Area mAU *s	Height [mAU]	Area %
1	1.516	BV	0.0556	15.55389	4.58715	0.2912
2	1.612	VV	0.0403	5.45407	2.11638	0.1021
3	1.796	VB	0.1393	19.05530	1.91724	0.3567
4	3.019	BB	0.0886	10.52565	1.76260	0.1971
5	10.097	BB	0.1140	5290.85791	703.05035	99.0529

Totals : 5341.44683 713.43372

*** End of Report ***
